# Development and Preliminary Validation of the Questionnaire (the First Edition) Based on TCM for Detecting Health Status in China

**DOI:** 10.1155/2015/863685

**Published:** 2015-10-11

**Authors:** Xuan Zhou, Fang Xu, Jian Gao, Shan Cao, Ziwei Zhao, Mingli Heng, Huaien Bu, Liqun Yin, Hongwu Wang

**Affiliations:** ^1^Chinese Internal Medicine, Graduate School, Tianjin University of Traditional Chinese Medicine, Anshan West Road 312, Nankai District, Tianjin 300193, China; ^2^Department of Public Health, Tianjin University of Traditional Chinese Medicine, Anshan West Road 312, Nankai District, Tianjin 300193, China; ^3^School of Humanistic Management, Tianjin University of Traditional Chinese Medicine, Tianjin 300073, China; ^4^Department of Mathematics, Tianjin University of Traditional Chinese Medicine, Anshan West Road 312, Nankai District, Tianjin 300193, China

## Abstract

*Background*. More and more people come to realize the importance of healthcare and early detecting of health status before becoming much more serious. Self-perceived health is an easy, economic, and effective indicator of health, which has been widely applied in measuring health. In this paper, the development and preliminary validation of the questionnaire (the First Edition) based on TCM theory were described and combined with Manual Mental Health Pattern for detecting health status in community of Tianjin, China. *Methods*. Questionnaire validity and reliability were evaluated in a small sample as a pilot study. Analyses included tests for reliability and internal consistency, exploratory factor analysis, and tests for discriminative ability and convergent validity. *Results*. Overall, 294 of 303 participants completed the questionnaire (97.3%). The questionnaire included 49 items. Cronbach's *α* was 0.83. Factor analysis established 10 distinct domains. The Pearson's rho correlation between the total scores and MHP (SCL) was statistically significant (*r* = 0.43, *P* < 0.001). *t*-test revealed significant differences (*P* < 0.05) in total scores between the healthy and unhealthy results distinguished by physical examination. *Conclusions*. Questionnaire reliability and validity were acceptable. Further work and larger sample would be warranted to refine items that measure the health status, to improve the reliability and discriminated validity of the questionnaire.

## 1. Introduction

With China entering the aging society, it is increasingly recognized that wellness and health promotion are important, as well as detecting health status to carry out early diagnosis and early treatment. Based on countries' progress and current new challenges in the field of traditional medicine, WHO Traditional Medicine Strategy 2014–2023 recently set out the course for TM and CM (T&CM) in the next decade. The key goals of the strategy are supporting Member States in harnessing the potential contribution of T&CM to health, wellness, and people-centered health care and promoting the safe and effective use of T&CM through the regulation of products, practices, and practitioners. One of the Strategic objectives is promoting universal health coverage by integrating T&CM services and self-health care into national health systems [1].

Chinese medicine theory has its special characteristic; many researchers paid attention to explore deep-seated theories and took it into practice, but more studies were concentrated in clinical treatment and drug development. The notion that prevention is better than cure was quite clear in Huangdi Neijing: Yellow Emperor's Canon of Medicine, the most important ancient Chinese medicine book. It is more valuable that monitoring and evaluating health status before he was ill. Moreover WHO defines health as a state of complete physical, mental, and social well-being and not merely the absence of disease or infirmity [2, 3]. Therefore we have attempted measuring health status by Chinese medicine theory [4–7]. In TCM theory, there are four diagnostic approaches that refer to inquiring, inspection, auscultation-olfaction, and palpation diagnosis and are regarded as basic in Chinese medicine. All these approaches are aimed at providing objective basis for differentiation of syndromes by collecting symptoms and signs from the patient. It is believed that by inspecting the exterior we can examine the interior viscera inside the body that can manifest themselves externally. Exterior means symptoms and signs that the disease reflects, while the interior means the fundamental pathology of the internal organs. According to this, theory of Chinese diagnosis, such as skin, complexion, smells, sounds, body build, and bones, can reflect the state of the internal organs. A practitioner of TCM can derive detailed information about the state of the whole organism, from examination of a small part of it [8].

## 2. Methods

### 2.1. Item Generation

Items were generated based on a review of the literature and through counseling with subject matter experts, including clinicians of TCM, mental health researchers, and scholars of TCM theory from provinces of Shandong and Jilin and cities of Tianjin and Shanghai and universities of Chinese Medicine and affiliated hospitals. Further discussions and modification of the items would be done after the pilot study and provided analysis results.

#### 2.1.1. Questionnaire of TCM and MHP

The questionnaire of TCM (the First Edition) included 49 items, which was designed for detecting the physical health status of the participants. 35 items were given indicative guidelines for frequency scores ranging from 0 (absent) to 1 (never), 2 (occasionally), 3 (sometimes), 4 (often), and 5 (always). 14 items included in the second part, scores were stand for 1 (no), 2 (yes).

We took MHP (Manual for Mental Health Pattern, Chinese version) in this study to measure the mental health state of the participants.

MHP, a scale that includes 40 items, classifies state of mental health as it pertains to stress and Quality of Life (QOL); the original version was developed by Japanese psychology professor Hashimoto Kimio in 1999, designed with six subscales to measure stress: Persistence, Lack of Concentration, Antisocial Behavior, Nervous Tension to Others, Fatigue, and Sleep/Wake up Disorder. Two subscales to measure QOL are as follows: Life Satisfaction and Life Passion. Each subscale consists of five items. There were 40 items and scores of each item were from 1 (not matched), 2 (not matched partly), and 3 (basically matched) to 4 (totally matched).

Four mental health patterns, Relaxed (standard stress adaptation), Energetic (stress adaptation), Fatigued (maladjustment) and Exhausted (stress disorder), were classified by using the Stress Check List (SCL) and QOL scores [9–11].

The Chinese version of MHP had been translated, revised, and standardized by professor Gaojian before the test that has been performed in more than 3000 people in Tianjin. The result was satisfactory, which was also consistent with psychometrics method [12]. The Chinese version scores of each item were added 4 (mostly matched), and 5 (totally matched).

The figure of score divided method and classification was attached in Appendix C.

### 2.2. Study Participants

The participants were selected from communities undergoing a regular physical examination in health examination center of Hospital in Tianjin. Participants had to meet the following inclusion criteria.

(1) They had to sign the informed consent before the interview, (2) their age was from 18 through 60 years, and (3) they lived in Tianjin city more than three year.

Participants were asked separately to complete the questionnaire. All participants underwent a standardized examination, including medical history, physical examination, blood hematology and biochemistry analysis, rest electrocardiography, and abdominal ultrasonography. A blinded assessor objectively measured the health status by the physical examination center.

### 2.3. Data Collection

After the questionnaire was completed, each participant was scheduled for physical examination in medical examination center. The completed questionnaire was checked by researchers to make sure all questions had been answered.

### 2.4. Statistical Analyses

Before analysis, all questionnaires were reread and checked for accuracy. All data were double entered with EPI DATA 3.1 (EpiData Association Odense, Denmark). The final dataset was converted into SPSS format. All statistical analyses were performed using the SPSS version 19.0 (SPSS, USA). Data were presented as percentages or means ± standard deviations (SD). Comparison between two groups was done with independent sample *t*-test.

### 2.5. Examination of Reliability and Validity Test

Internal consistency is a measure of reliability that assesses the degree to which the items were related to each other, it measures a unified construct [13]. Internal consistency was measured with Cronbach's alpha (*α*).

Exploratory Factor Analysis (EFA) was used to determine the scale of the items mainly due to the TCM theory aspects; principal component exploratory factor analysis with varimax rotation with Kaiser normalization was carried out to assess the underlying structure of questionnaire items [14]. The criterion applied to retain scales was an eigen value ≥ 1.0 for that scale [15]. The critical threshold for each item to meet this condition has been preset at 0.30. After determining scales, internal consistency was retested by calculating Cronbach's *α* coefficient for subscales.

Criterion-related validity of the questionnaire was assessed with Pearson's rho correlation coefficients between the scores of the questionnaire and somatic stress dimension of the MHP [16]. It hypothesized that the scores would significantly correlate with the scores for stress. *t*-tests used to determine whether the questionnaire was able to distinguish between healthy and unhealthy status as measured by the physical examination results in hospital and psychological measurement by MHP. The differences between group comparisons were determined using analysis of *t*-test, when *P* value lower than 0.05 was considered as statistically significant.

## 3. Results

### 3.1. Characteristics of Participants

294 of 303 participants completed the questionnaire (97.03%). The data of 294 people were collected, female were 129 (43.88%), and male were 165 (56.12%). Mean age of participants was 41.35 (standard deviation 8.57).

### 3.2. Mental Health State of Participants: MHP Scores

The mental health state of participants and two genders were shown in Table 1. The mean scores of QOL (36.43) and SCL (45.07) of study participants located the point in the number 2 area of Cartesian coordinates. It showed that participants were in the state of Relaxed, Standard stress adaptation, which indicated that their mental health states were in a good condition. There was significant difference between scores of genders in scales: Lack of Concentration, Nervous Tension to Others, Fatigue, Social Stress, and Somatic Stress. It indicated that women were more liable to feel stress than men.

### 3.3. Internal Consistency

Internal consistency results using the Cronbach's *α* coefficient, 49 items were 0.83 for the whole questionnaire (see Table 2); Cronbach's *α* higher than 0.6 was acceptable [17] and Cronbach's *α* coefficient of scale VII to X was below our desired value; although we decided to retain this scale, further modification would be made of these scales and items.

### 3.4. Factor Analysis

The Kaiser-Meyer-Olkin measure of sampling adequacy (KMO) was 0.64, and the Bartlett test of sphericity was significant (*χ* ^2^(1176) = 4888.12, *P* < 0.001), indicating that the data were suitable for factor analysis. 16 factors had eigen values > 1, explaining 65.17% of the total variance. By scree test and TCM theory conclusion, 10-factor solution was more interpretable. 10 factors explained 50.15% of the total variance. Each factor and loadings of the items are provided in Table 3. As shown in the table, the ten factors were (1) heart system (11 items), (2) spleen and stomach system (5 items), (3) lung system (5 items), (4) urine and stool (4 items), (5) metabolic systems (4 systems), (6) liver system (5 items), (7) head (4 items), (8) body (4 items), (9) kidney system (4 items), and (10) skin (3 items). Cronbach's *α* and intercorrelation of Subscales were showed in Tables 2 and 4.

### 3.5. Discriminative Ability

*t*-test revealed significant differences (*t* = −4.21, *P* = 0.000) in total scores, between the healthy (57.23 ± 6.00) and unhealthy (62.65 ± 11.03) results distinguished by physical examination. As presented in Table 5, the score of the questionnaire did not differ significant (*t* = −0.43, *P* = 0.67) between males (60.93 ± 10.89) and females (61.59 ± 9.66). Score of female in healthy (56.31 ± 5.20) and unhealthy status (63.60 ± 10.22) showed significant differences (*t* = −4.55, *P* = 0.000), but male did not indicate significant differences (*t* = −1.23, *P* = 0.22).

### 3.6. Convergent Validity

The correlation between the score of questionnaire (60.63 ± 9.55) and that for SCL of MHP (44.35 ± 13.60, Cronbach's *α* = 0.74) was statistically significant (Pearson's *r* = 0.43, *P* < 0.001). Scores of somatic stress subscale of MHP and the questionnaire showed significant differences (Pearson's *r* = 0.53, *P* < 0.001).

## 4. Discussion

294 of 303 participants completed the questionnaire (97.3%), indicating that it is user-friendly and easily understand for participants, and they responded to the questions carefully. In this pilot study, Cronbach's alpha is 0.83, which shows a good level of internal consistency for the questionnaire, as reliability coefficients were evaluated according to Nunnally and Bernstein [18] (*α* > 0.70 = acceptable, *α* > 0.80 = good, and *α* > 0.90 = excellent).

The traditional Chinese medicine considers that various factors can affect health, from physical, psychological, nature, and society, and these factors influence each other [19].

Because healthy state should be of complete physical, mental, and social well-being, the study utilized the MHP Scale to measure the mental health state of the participants. The results showed that the questionnaire was able to discriminate between groups. As expected, the correlation between the score of questionnaire scores of the questionnaire and SCL of MHP and somatic stress subscale scores were statistically significant differences. The higher scores represent a less healthy mental, social, and physical state of human body. They have a good consistency.

When individual internal consistency was analyzed further within each domain, Cronbach's *α* for VII to X subscales (head, body, skin, and kidney system) was relatively low (0.40 to 0.52). This could be due to the small number of items for each subscale and most of the items are two-graded. Further modifications should be made to refine the items number and contents, some items probably are to be removed in the formal investigation, and make the questionnaire much more succinct.

In health care, many of the variables are abstract concepts known as theoretical constructs. Using tests or instruments is valid and reliable to measure such constructs [20]. Through the factor analysis, the underlying dimensions could be extracted to support this conceptual model. The analysis resulted in 10 distinct factors, as conceptualized in this model. However, the first factor consisted of 11 items that seemed to describe 2 dimensions, including symptoms of kidney and skin. Cronbach's *α* of the scales was 0.40 and 0.44, respectively, lower than that for the domain of heart system (0.77). Therefore, the study did not regroup these items into 2 groups. In the light of the traditional Chinese medical theory regarding the human body as a whole, themes of holism are deeply embedded in the doctrine of TCM [21, 22].

## 5. Limitations

Some symptoms may appear in different dimensions, taking the symptom of edema as example, which may occur both in heart and kidney system. As a result, it leads to collinearity, which has influence on stability of the dimension. That may cause factor analysis of the overall result that is not ideal, though it can still reflect the basic structure of the questionnaire in accordance with the theory of TCM. In other examples, the morbidity of the organs can be revealed by the human facial complexion [23–27], and symptom of fatigue may occur not only in heart disease but also in liver or endocrine problems such as diabetes [28–30]. These give guidance for the follow-up study to structure optimization and adjusting; future research could explore performing a confirmatory factor analysis of these results. Many complementary medicine researchers confront the same research design problems such as the spectrum of interventions, holistic concepts, and individual practices [31–34], but more and more studies try to explore the right points of combining the ancient TCM theories with constantly changing environment, social-demographic, reproductive, lifestyle, systemic health, emotional status, and so on [35–37].

The participants were selected from communities that attended the physical examination in health examination center of Hospital, by convenience sampling which is widely used in health-related scales [38, 39]. Nevertheless, the imperfection of the sample is less representative than a random sample and may limit the generalizability of this study. Therefore, it would be valuable to test the questionnaire on a representative larger sample in more places of Tianjin at next stage of studies.

## 6. Conclusion

The questionnaire of TCM (first version) was established for detecting physical health status; it is easy to complete, for applying in community health care. The 49-item questionnaire encompasses the domains of heart, spleen and stomach, lung, endocrine and metabolic syndrome, liver, kidney system, head, body, skin, urine, and stool feces.

Although more work is needed in further refinement of the structure, it will be useful in moving towards developing the integrating T&CM services and self-health care into national health systems.

## Figures and Tables

**Figure 1:**
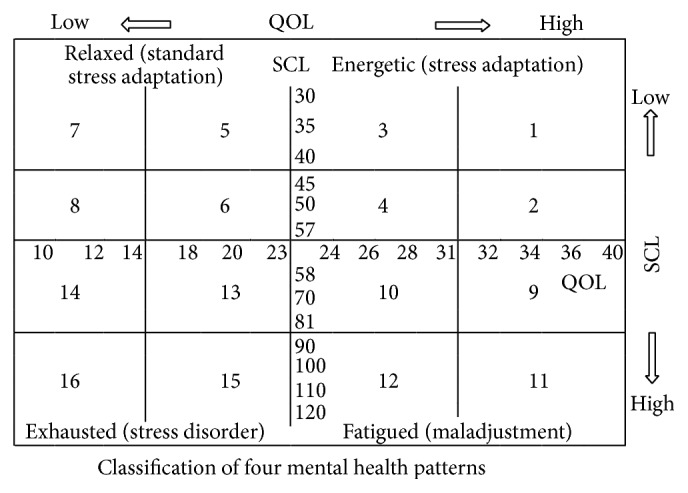


**Table 1:** MHP scores of participants and two genders in study.

MHP subscales	Participants (*n* = 294)		Male (*n* = 165)		Female (*n* = 129)		*t* value	*P*
	Mean	SD	Mean	SD	Mean	SD		
Psychological Stress								
Persistence	7.11	2.89	7.01	2.94	7.23	2.82	−0.65	0.516
Lack of Concentration	7.28	2.81	6.88	2.22	7.77	3.35	−2.70^*∗*^	0.007

Social Stress								
Antisocial Behavior	6.82	2.79	6.54	2.40	7.19	3.19	−1.97^*∗*^	0.049
Nervous Tension to Others	7.78	2.80	7.36	2.27	8.31	3.29	−2.89^*∗*^	0.004

Somatic Stress								
Fatigue	7.79	3.29	7.38	3.14	8.32	3.42	−2.44^*∗*^	0.015
Sleep/Wake up Disorder	7.89	3.71	7.56	3.65	8.29	3.76	−1.66	0.098

QOL								
Life Satisfaction	19.28	5.75	19.36	5.84	19.18	5.65	0.26	0.796
Life Passion	16.79	4.34	16.98	4.45	16.55	4.22	0.83	0.406

Psychological Stress	14.29	5.17	13.77	4.59	14.95	5.78	−1.94	0.054
Social Stress	14.49	4.99	13.76	4.21	15.43	5.72	−2.87^*∗*^	0.004
Somatic Stress	15.57	6.18	14.80	6.03	16.55	6.25	−2.41^*∗*^	0.017

SCL	44.35	13.60	42.33	12.11	46.92	14.94	−2.90^*∗*^	0.004
QOL	35.84	9.67	36.02	9.99	35.60	9.26	0.37	0.712

^*∗*^ *P* < 0.05.

**Table 2:** Cronbach's *α* coefficient of questionnaire and 10 subscales.

Subscale	Number of items	Cronbach's *α*
Questionnaire	49	0.83
I	11	0.77
II	5	0.75
III	5	0.73
IV	4	0.65
V	4	0.68
VI	5	0.63
VII	4	0.40
VIII	4	0.52
IX	4	0.47
X	3	0.44

**Table 3:** Exploratory factor analysis of questionnaire.

Items, subscales, and total	EFA factor loadings										Communalities
	Factor 1	Factor 2	Factor 3	Factor 4	Factor 5	Factor 6	Factor 7	Factor 8	Factor 9	Factor 10	
*(I) Heart system *											
Palpitation	**0.74 **	0.18	0.02	0.00	0.10	0.16	0.11	0.14	0.03	−0.09	0.66
Shortness of breath	**0.62 **	0.25	0.10	−0.08	0.11	0.00	−0.16	−0.11	−0.07	0.12	0.53
Chest distress	**0.60 **	0.12	0.11	−0.14	0.07	0.13	0.07	0.06	0.11	−0.18	0.49
Vexation	**0.57 **	0.14	0.04	0.04	0.22	0.34	0.02	0.14	−0.07	−0.07	0.54
Dizziness	**0.55 **	0.05	0.01	0.30	0.11	0.11	0.01	0.10	−0.06	0.03	0.44
Memory deterioration	**0.52 **	−0.11	−0.03	0.11	−0.06	0.13	0.10	0.00	0.27	0.03	0.40
Edema	**0.48 **	−0.07	−0.11	0.19	−0.13	0.00	0.12	−0.05	0.19	0.18	0.39
Lassitude in the knees	**0.46 **	0.01	0.05	0.12	0.17	−0.23	0.35	−0.13	0.22	0.07	0.50
Fatigue	**0.40 **	0.10	0.16	−0.01	0.38	−0.07	0.06	−0.10	−0.07	0.16	0.38
Soreness of the waist	**0.39 **	−0.01	0.06	−0.08	0.19	0.04	0.19	−0.12	0.34	−0.14	0.39
Skin swelling	**0.36 **	−0.12	−0.12	0.14	−0.11	−0.15	0.00	−0.03	0.09	0.33	0.33

*(II) Spleen and stomach system *											
Vomit	−0.02	**0.81 **	0.01	0.03	0.11	0.02	−0.04	0.13	0.11	0.03	0.69
Belching	0.14	**0.75 **	0.00	−0.01	−0.05	−0.05	0.01	−0.04	0.06	−0.02	0.59
Nausea	−0.02	**0.74 **	−0.01	0.00	0.15	−0.01	0.02	0.09	0.21	0.00	0.63
Abdominal distension	0.22	**0.62 **	0.06	−0.02	−0.06	0.04	0.24	0.04	−0.10	−0.01	0.51
Acid regurgitation	0.07	**0.55 **	0.07	0.20	−0.01	0.05	0.39	−0.07	−0.23	0.07	0.58

*(III) Lung system *											
Nasal obstruction	−0.01	−0.11	**0.72 **	−0.02	−0.10	0.04	−0.12	0.04	−0.13	−0.08	0.58
Cough	0.11	0.10	**0.70 **	−0.05	0.07	−0.05	0.23	−0.01	0.21	−0.02	0.61
Running nose	0.01	−0.04	**0.70 **	−0.01	−0.06	0.16	−0.07	0.22	−0.10	0.03	0.58
Expectoration	0.04	0.12	**0.63 **	−0.03	0.00	0.08	0.28	0.05	0.23	0.13	0.57
Throat itching	0.02	0.10	**0.62 **	0.22	0.14	−0.05	−0.05	−0.07	0.07	−0.05	0.48

*(IV) Urine and stool *											
Dysuria	−0.06	0.05	0.02	**0.81 **	0.07	0.13	−0.06	0.01	0.00	−0.07	0.69
Urgency of urination	0.27	−0.14	−0.04	**0.67 **	0.08	−0.07	0.16	−0.05	0.16	−0.05	0.61
Frequency of micturition	0.15	−0.13	0.33	**0.64 **	0.14	−0.07	0.04	−0.05	−0.01	−0.01	0.58
Diarrheas	−0.02	0.19	−0.05	**0.63 **	−0.08	0.12	−0.09	0.04	−0.01	0.08	0.47

*(V) Endocrine & Metabolic system *											
Overdrinking	0.03	−0.05	−0.05	−0.02	**0.70 **	−0.05	−0.06	−0.02	−0.10	0.14	0.53
Dryness of mouth	0.21	0.11	0.09	0.21	**0.69 **	0.07	0.02	0.06	−0.01	−0.06	0.60
Polydipsia	0.07	0.19	−0.02	0.19	**0.69 **	0.11	0.05	0.09	0.09	−0.14	0.60
Polyuria	0.01	−0.07	−0.01	−0.10	**0.65 **	0.00	0.08	−0.05	0.06	−0.03	0.45

*(VI) Liver system *											
Insomnia, dream disturbed sleep	0.05	−0.04	−0.04	0.15	0.12	**0.71 **	0.06	−0.08	0.29	0.14	0.66
Pruritus	0.29	−0.13	−0.08	0.21	0.12	**0.64 **	0.11	−0.06	0.34	0.14	0.72
Neck symptoms	0.18	0.10	0.03	−0.04	−0.13	**0.49 **	−0.08	−0.01	−0.17	0.08	0.35
Pain	0.30	0.14	0.11	−0.03	−0.04	**0.44 **	0.19	−0.04	0.19	−0.17	0.42
Symptoms of bleeding	−0.02	−0.06	0.14	0.05	0.04	**0.40 **	0.20	−0.08	−0.10	0.05	0.24

*(VII) Head *											
Five sense organs	0.12	0.05	0.05	0.00	−0.01	−0.02	**0.64 **	−0.01	0.10	−0.01	0.45
Throat	0.08	0.10	0.17	0.14	−0.01	0.10	**0.53 **	0.03	−0.06	0.11	0.37
Mouth (bad breath)	−0.08	−0.01	−0.01	−0.10	0.09	0.16	**0.48 **	0.01	0.17	−0.21	0.35
Teeth and gums	0.11	0.03	−0.15	−0.10	0.05	0.09	**0.40 **	0.13	0.03	0.14	0.25

*(VIII) Body *											
Waist and back	−0.02	0.08	0.09	−0.01	0.05	−0.08	−0.01	**0.90 **	0.09	0.02	0.83
Chest and abdomen	−0.07	−0.09	0.09	0.00	0.02	−0.09	−0.09	**0.70 **	0.12	0.10	0.56
Hoarse voice or aphonia	0.06	0.20	−0.07	−0.01	0.00	−0.07	0.27	**0.45 **	−0.05	−0.11	0.34
Renal percussive pain	0.25	0.09	0.05	−0.02	−0.09	0.15	0.21	**0.40 **	−0.21	−0.08	0.36

*(IX) Kidney system *											
Hypoacusis	0.04	0.28	0.04	−0.09	0.10	−0.05	0.29	0.04	**0.56 **	0.02	0.50
Arms and legs	0.19	0.04	0.01	0.08	−0.09	−0.07	−0.06	0.34	**0.49 **	0.03	0.42
Tinnitus	0.06	0.01	0.06	0.11	−0.05	0.12	0.04	0.00	**0.43 **	0.15	0.24
Simultaneous sweat and night sweat	0.19	0.38	0.12	−0.14	0.05	0.30	−0.19	−0.05	**0.42 **	−0.09	0.54

*(X) Skin *											
Complexion	0.03	0.03	−0.02	−0.05	0.05	0.05	0.08	−0.05	0.05	**0.79 **	0.64
Skin color and luster	−0.01	0.01	−0.02	0.00	−0.01	0.11	−0.07	0.02	0.12	**0.75 **	0.59
Skin diseases	−0.14	0.05	0.15	−0.07	−0.02	0.21	0.17	0.24	−0.13	**0.38 **	0.34

Eigen values	3.72	3.16	2.64	2.45	2.40	2.15	2.12	2.09	1.95	1.88	
% of variance	7.60	6.45	5.39	5.00	4.91	4.38	4.33	4.27	3.98	3.85	
Cumulative %	7.60	14.05	19.44	24.45	29.35	33.73	38.06	42.33	46.31	50.15	

Extraction method: principal component analysis. Varimax rotation with Kaiser normalization, sorted by size.

Bold font for the EFA factor loadings indicates the scale on which the items load.

**Table 4:** Intercorrelation of subscales (Pearson's *r*).

Subscale	Factor 1	Factor 2	Factor 3	Factor 4	Factor 5	Factor 6	Factor 7	Factor 8	Factor 9	Factor 10
I	1									
II	0.25^*∗∗*^	1								
III	0.16^*∗*^	0.13^*∗*^	1							
IV	0.24^*∗∗*^	0.05	0.13^*∗*^	1						
V	0.32^*∗∗*^	0.14^*∗*^	0.08	0.19^*∗*^	1					
VI	0.41^*∗∗*^	0.11^*∗*^	0.13^*∗*^	0.18^*∗*^	0.15^*∗*^	1				
VII	0.27^*∗∗*^	0.23^*∗∗*^	0.10	0.05	0.08	0.25^*∗∗*^	1			
VIII	0.13^*∗*^	0.19^*∗*^	0.14^*∗*^	−0.02	0.05	0.02	0.13^*∗*^	1		
IX	0.33^*∗∗*^	0.26^*∗∗*^	0.17^*∗*^	0.06	0.12	0.28^*∗∗*^	0.17^*∗*^	0.10	1	
X	0.00	0.06	0.07	−0.03	−0.03	0.11	0.16^*∗*^	0.03	0.10	1

^*∗*^ *P* < 0.05, ^*∗∗*^ *P* < 0.001.

**Table 5:** Scores of questionnaire in different physical exam results and genders.

Physical exam result	Gender				Total	*t* value	*P*
	Female		Male				
	*n*	Scores mean ± SD	*n*	Scores mean ± SD			
Healthy	26	56.31 ± 5.20	21	58.38 ± 6.83	47	1.18	0.24
Unhealthy	68	63.60 ± 10.22	69	61.71 ± 11.78	137	−1.00	0.32
Total	94	61.59 ± 9.66	90	60.93 ± 10.89	184^*∗*^	−0.43	0.67

^**∗**^There were 110 of 294 participants that were excluded, because their physical examination results were subhealth status.

**Table 6:** 

Do you have the symptoms of the following? How often?						
		1	2	3	4	5
1	Nasal obstruction	□	□	□	□	□
2	Running nose	□	□	□	□	□
3	Throat itching	□	□	□	□	□
4	Cough	□	□	□	□	□
5	Expectoration	□	□	□	□	□
6	Chest distress	□	□	□	□	□
7	Palpitation	□	□	□	□	□
8	Vexation	□	□	□	□	□
9	Dryness of mouth	□	□	□	□	□
10	Polydipsia	□	□	□	□	□
11	Acid regurgitation	□	□	□	□	□
12	Belching	□	□	□	□	□
13	Nausea	□	□	□	□	□
14	Vomit	□	□	□	□	□
15	Abdominal distension	□	□	□	□	□
16	Diarrhea	□	□	□	□	□
17	Soreness of the waist	□	□	□	□	□
18	Lassitude in the knees	□	□	□	□	□
19	Frequency of micturition	□	□	□	□	□
20	Urgency of urination	□	□	□	□	□
21	Dysuria	□	□	□	□	□
22	Polyuria	□	□	□	□	□
23	Edema	□	□	□	□	□
24	Fatigue	□	□	□	□	□
25	Shortness of breath	□	□	□	□	□
26	Simultaneous sweat and night sweat	□	□	□	□	□
27	Overdrinking	□	□	□	□	□
28	Dizziness	□	□	□	□	□
29	Memory deterioration	□	□	□	□	□
30	Insomnia, dream disturbed sleep	□	□	□	□	□
31	Hypoacusis	□	□	□	□	□
32	Tinnitus	□	□	□	□	□
33	Pain	□	□	□	□	□
34	Symptoms of bleeding	□	□	□	□	□
35	Pruritus	□	□	□	□	□

Physical examination: Is there something wrong with the part of body as following?						
		YES	NO	Comments		

36	Complexion	□	□			
37	Skin color and luster	□	□			
38	Skin diseases	□	□			
39	Skin swelling	□	□			
40	Five sense organs: eyes, ears, nose, and lips	□	□			
41	Teeth and gums	□	□			
42	Mouth (bad breath)	□	□			
43	Hoarse voice or aphonia	□	□			
44	Throat	□	□			
45	Neck	□	□			
46	Chest and abdomen	□	□			
47	Waist and back	□	□			
48	Arms and legs	□	□			
49	Renal percussive pain	□	□			

**Table 7:** 

Items	Correlation		Cronbach's *α*	Low score group (*n* = 82)		High score group (*n* = 81)			*t* value
	(Pearson's *r*)			$\overset{-}{\chi }$	*s*	$\overset{-}{\chi }$	*s*		
Nasal obstruction	0.13	*∗*	0.83	1.12	0.46	1.38	0.78	−2.59	*∗∗*
Running nose	0.24	*∗∗∗*	0.83	1.04	0.19	1.46	0.85	−4.33	*∗∗∗*
Throat itching	0.33	*∗∗∗*	0.83	1.12	0.43	1.69	1.11	−4.30	*∗∗∗*
Cough	0.44	*∗∗∗*	0.82	1.12	0.48	1.85	1.07	−5.59	*∗∗∗*
Expectoration	0.44	*∗∗∗*	0.82	1.04	0.25	1.81	1.04	−6.57	*∗∗∗*
Chest distress	0.49	*∗∗∗*	0.82	1.05	0.27	1.78	0.95	−6.66	*∗∗∗*
Palpitation	0.61	*∗∗∗*	0.82	1.01	0.11	1.89	0.96	−8.15	*∗∗∗*
Vexation	0.55	*∗∗∗*	0.82	1.02	0.22	1.91	0.90	−8.66	*∗∗∗*
Dryness of mouth	0.46	*∗∗∗*	0.82	1.02	0.16	1.93	1.01	−7.94	*∗∗∗*
Polydipsia	0.40	*∗∗∗*	0.82	1.00	0.00	1.63	0.94	−6.02	*∗∗∗*
Acid regurgitation	0.34	*∗∗∗*	0.82	1.00	0.00	1.46	1.00	−4.11	*∗∗∗*
Belching	0.31	*∗∗∗*	0.82	1.00	0.00	1.19	0.67	−2.48	*∗*
Nausea	0.31	*∗∗∗*	0.82	1.01	0.11	1.23	0.64	−3.09	*∗∗*
Vomit	0.30	*∗∗∗*	0.83	1.00	0.00	1.60	0.58	−2.49	*∗*
Abdominal distension	0.38	*∗∗∗*	0.82	1.01	0.11	1.42	0.92	−3.96	*∗∗∗*
Diarrhea	0.17	*∗∗*	0.83	1.01	0.11	1.16	0.56	−2.38	*∗*
Soreness of the waist	0.47	*∗∗∗*	0.82	1.12	0.46	2.12	1.18	−7.15	*∗∗∗*
Lassitude in the knees	0.44	*∗∗∗*	0.82	1.01	0.11	1.48	0.96	−4.36	*∗∗∗*
Frequency of micturition	0.31	*∗∗∗*	0.82	1.00	0.00	1.26	0.76	−3.09	*∗∗*
Urgency of urination	0.33	*∗∗∗*	0.82	1.02	0.22	1.26	0.72	−2.81	*∗∗*
Dysuria	0.19	*∗∗*	0.83	1.00	0.00	1.06	0.37	−1.52	
Polyuria	0.20	*∗∗*	0.83	1.00	0.00	1.20	0.75	−2.38	*∗*
Edema	0.35	*∗∗∗*	0.82	1.04	0.25	1.73	1.05	−5.78	*∗∗∗*
Fatigue	0.42	*∗∗∗*	0.82	1.06	0.36	1.73	0.98	−5.78	*∗∗∗*
Shortness of breath	0.42	*∗∗∗*	0.82	1.01	0.11	1.53	0.82	−5.62	*∗∗∗*
Simultaneous sweat and night sweat	0.41	*∗∗∗*	0.82	1.01	0.11	1.57	0.94	−5.32	*∗∗∗*
Overdrinking	0.15	*∗*	0.83	1.00	0.00	1.15	0.67	−1.98	
Dizziness	0.48	*∗∗∗*	0.82	1.07	0.31	1.85	0.96	−6.94	*∗∗∗*
Memory deterioration	0.43	*∗∗∗*	0.82	1.09	0.32	1.73	0.94	−5.85	*∗∗∗*
Insomnia, dream disturbed sleep	0.44	*∗∗∗*	0.82	1.05	0.22	2.32	1.29	−8.74	*∗∗∗*
Hypoacusis	0.34	*∗∗∗*	0.82	1.01	0.11	1.28	0.66	−3.68	*∗∗∗*
Tinnitus	0.25	*∗∗∗*	0.83	1.01	0.11	1.37	0.87	−3.67	*∗∗∗*
Pain	0.53	*∗∗∗*	0.82	1.40	0.72	3.05	1.07	−11.52	*∗∗∗*
Symptoms of bleeding	0.25	*∗∗∗*	0.83	1.17	0.41	1.78	1.04	−4.90	*∗∗∗*
Pruritus	0.56	*∗∗∗*	0.82	1.23	0.53	2.84	1.12	−11.62	*∗∗∗*
Complexion	0.11		0.83	1.00	0.00	1.07	0.26	−2.53	*∗*
Skin color and luster	0.08		0.83	1.00	0.00	1.04	0.19	−1.75	
Skin diseases	0.09		0.83	1.04	0.19	1.11	0.32	−1.82	
Skin swelling	0.12	*∗*	0.83	1.00	0.00	1.04	0.19	−1.75	
Five sense organs: eyes, ears, nose, and lips	0.29	*∗∗∗*	0.83	1.02	0.16	1.22	0.42	−3.99	*∗∗∗*
Teeth and gums	0.21	*∗∗∗*	0.83	1.45	0.50	1.72	0.45	−3.54	*∗∗∗*
Mouth (bad breath)	0.17	*∗∗*	0.83	1.01	0.11	1.07	0.26	−1.95	
Hoarse voice or aphonia	0.12	*∗*	0.83	1.00	0.00	1.02	0.16	−1.42	
Throat	0.33	*∗∗∗*	0.82	1.02	0.16	1.28	0.45	−4.88	*∗∗∗*
Neck	0.20	*∗∗∗*	0.83	1.04	0.19	1.20	0.40	−3.27	*∗∗*
Chest and abdomen	0.01		0.83	1.00	0.00	1.00	0.00	−1.00	
Waist and back	0.09		0.83	1.00	0.00	1.01	0.11	−1.00	
Arms and legs	0.24	*∗∗∗*	0.83	1.00	0.00	1.17	0.38	−4.09	*∗∗∗*
Renal percussive pain	0.22	*∗∗∗*	0.83	1.01	0.11	1.09	0.28	−2.20	*∗*

*t* value: independent-sample test. *r*: the correlation coefficient of item and the total score.

It is divided into two groups according to the scores: a high mark group and a low mark group. 27% of the participants in high score sorted into the high score group, and 27% who got the low scores entered the low score group. *∗∗∗*: *P* < 0.001, *∗∗*: *P* < 0.01, *∗*: *P* < 0.05.

**Table 8:** Construction of the MHP Scale.

MHP subscales		Items (number)
Psychological Stress	Persistence	1, 9, 17, 25, 33
	Lack of Concentration	2, 10, 18, 26, 34

Social Stress	Antisocial Behavior	3, 11, 19, 27, 35
	Nervous Tension to Others	4, 12, 20, 28, 36

Somatic Stress	Fatigue	5, 13, 21, 29, 37
	Sleep/Wake up Disorder	6, 14, 22, 30, 38

QOL	Life Satisfaction	7, 15, 23, 31, 39
	Life Passion	8, 16, 24, 32, 40

SCL	Psychological Stress + Social Stress + Somatic Stress	

## Appendices

### A. Questionnaire: 49 Items

The questions in Table 6 inquire about health events during the last 2 weeks. Answer every question by marking the appropriate box with a “✓.” You may choose from one of the following answers:

1: never or almost never
2: occasionally
3: often
4: very often
5: always

### B. Items Analysis Results

See Table 7.

### C. MHP Scale: Construction and Classification Method

See Table 8 and Figure 1.
